# XGBoost Enhances the Performance of SAFE: A Novel Microwave Imaging System for Early Detection of Malignant Breast Cancer

**DOI:** 10.3390/cancers17020214

**Published:** 2025-01-10

**Authors:** Ali Yurtseven, Aleksandar Janjic, Mehmet Cayoren, Onur Bugdayci, Mustafa Erkin Aribal, Ibrahim Akduman

**Affiliations:** 1 Mitos Medical Technologies, ITU Ayazaga Ari 1, Maslak, 34469 Istanbul, Turkey; aleksandarjanjic@mitosmedikal.com (A.J.); mehmet.cayoren@gmail.com (M.C.); erkin.aribal@acibadem.com (M.E.A.); akduman@itu.edu.tr (I.A.); 2 Electrical and Electronics Engineering Faculty, Istanbul Technical University, Maslak, 34469 Istanbul, Turkey; 3 Department of Radiology, School of Medicine, Marmara University, Pendik, 34899 Istanbul, Turkey; onurbug@hotmail.com; 4 Radiology Department, Breast Health Center, Altunizade Hospital, Acibadem M.A.A. University, Atasehir, 34684 Istanbul, Turkey

**Keywords:** SAFE, microwave imaging, breast cancer detection, machine learning, medical imaging, tumor classification

## Abstract

Background/Objectives: Breast cancer is a significant global health concern, and early detection is crucial for improving patient outcomes. Mammography is widely used but has limitations, particularly for younger women with denser breasts. These include reduced sensitivity, false positives, and radiation risks. This highlights the need for alternative screening methods. In this study, we assess the performance of SAFE (Scan and Find Early), a novel microwave imaging device, in detecting breast cancer in a larger patient cohort. Unlike previous studies that predominantly relied on cross-validation, this study employs a more reliable, independent evaluation methodology to enhance generalizability. Methods: We developed an XGBoost model to classify breast cancer cases into positive (malignant) and negative (benign or healthy) groups. The model was analyzed with respect to key factors such as breast size, density, age, tumor size, and histopathological findings. This approach provides a better understanding of how these factors influence the model’s performance, using an independent evaluation methodology for increased reliability. Results: Our results demonstrate that SAFE exhibits high sensitivity, particularly in dense breasts (91%) and younger patients (83%), suggesting its potential as a supplemental screening tool. Additionally, the system shows high detection accuracy for both small (<2 cm) and larger lesions, proving effective in early cancer detection. Conclusions: This study reinforces the potential of SAFE to complement existing screening methods, particularly for patients with dense breasts, where mammography’s sensitivity is reduced. The promising results warrant further research to solidify SAFE’s clinical application as an alternative screening tool for breast cancer detection.

## Simple Summary

Breast cancer is one of the most common cancers affecting women worldwide, and early detection is key to improving survival rates. However, current screening methods, like mammography, face limitations, particularly in younger women and those with dense breast tissue, where sensitivity is reduced, and there is a risk of false positives and radiation exposure. To address these challenges, we developed SAFE (Scan and Find Early), a microwave imaging device that uses non-ionizing electromagnetic waves to detect breast cancer safely and effectively. In this study, we evaluated SAFE on a large patient cohort, analyzing its ability to classify cases into malignant (cancerous) and non-cancerous (benign or healthy) categories. SAFE demonstrated high sensitivity, especially in younger patients (83%) and those with dense breasts (91%). It also successfully detected both small (<2 cm) and larger tumors, highlighting its potential for early diagnosis. Unlike traditional methods, SAFE’s innovative approach offers a safer, radiation-free alternative, making it particularly suitable for frequent screenings in younger and high-risk populations. Our findings show that SAFE could complement existing tools, improving breast cancer detection and reducing diagnostic uncertainties. With further research, SAFE could play a pivotal role in transforming breast cancer screening and saving lives.

## 1. Introduction

Breast cancer is the most common form of cancer among women and remains the leading cause of cancer-related deaths in women globally [1]. For a long time, researchers have been developing diagnostic tools and promising treatments for the disease. Studies emphasize the importance of early diagnosis of tumor lesions to improve treatment success rates. Consequently, it is crucial to develop innovative diagnostic technologies that can efficiently detect tumor cells before their progression [2].

While significant advancements in breast cancer treatment strategies have contributed to a reduction in mortality rates, early detection through mammographic screening remains a key tool in improving survival outcomes [3]. However, mammography comes with several challenges, some of which limit its effectiveness, especially in certain populations [4,5].

One limitation of mammography is that it presents unique challenges for younger women, particularly those with a family history of breast cancer. Women under 50, especially those with dense breast tissue, experience reduced sensitivity and specificity in mammography, making it more difficult to detect small tumors [6,7,8,9]. The increased density of younger women’s breasts makes it harder to distinguish tumors from the surrounding tissue, leading to false negatives [10,11,12,13,14,15,16]. Moreover, these younger women often experience a higher rate of false positives, which can lead to unnecessary follow-up tests, biopsies, and emotional distress [17,18]. One study found that over a nine-year period of annual mammograms, the likelihood of a false positive result for women aged 40–69 years was 43%, with the rate increasing to nearly 100% for women with a family history of breast cancer or other risk factors, such as benign breast disease or hormone use. This is particularly concerning, as it can lead to unnecessary treatments and anxiety, sometimes exposing women to unnecessary procedures and psychological burdens [17].

Mammography also carries risks due to the radiation exposure associated with repeated screenings [4,19]. Younger women are more susceptible to radiation-induced cancers due to their increased cell division rates, making them more vulnerable to the carcinogenic effects of ionizing radiation. This is particularly relevant for women under 50 or those with a family history of breast cancer, who may already be genetically predisposed to developing the disease [20]. Studies have shown that the cumulative radiation risk over many years of mammographic screening may outweigh the benefits, especially for those with genetic risks [21]. For women with inherited mutations such as BRCA1 or BRCA2, the risk of radiation-induced breast cancer may be higher, and early screening may not always lead to a significant reduction in mortality [21,22]. Additionally, while mammography may detect cancer at an earlier stage, no conclusive evidence exists to show that early mammography in these younger, high-risk groups leads to a decrease in breast cancer mortality [23].

Another challenge is the issue of overdiagnosis. Mammography can detect both invasive cancers and ductal carcinoma in situ (DCIS), a non-invasive form of cancer. However, some of these cancers may never grow or spread, leading to overdiagnosis and overtreatment [24]. Overdiagnosis occurs when cancers that would not have caused harm are treated unnecessarily, exposing patients to the risks and side effects of treatments like surgery, chemotherapy, or radiation. This issue is particularly relevant in younger women, where the detection of slow-growing, non-aggressive cancers could lead to unnecessary interventions that offer little benefit [25].

Furthermore, while contrast-enhanced mammography (CEM) can improve detection rates in dense breast tissue, it carries its own set of limitations and risks. CEM involves the use of contrast agents to highlight areas of abnormal blood supply, but this method increases radiation exposure compared to conventional mammography. Although it improves the sensitivity of mammograms in detecting tumors in dense breasts, the added radiation dose may be concerning, particularly for younger women and those undergoing multiple imaging studies over time. Additionally, CEM is associated with allergic reactions to the contrast agents, as well as renal toxicity in patients with impaired kidney function. The increased cost, limited accessibility, and potential adverse effects of contrast agents also make CEM less practical for widespread adoption, particularly in resource-limited settings [26,27,28].

Given these limitations, it is clear that mammography alone may not be sufficient for optimal breast cancer detection, especially for high-risk groups such as younger women, those with dense breast tissue, or women with a family history of breast cancer. This highlights the urgent need for alternative technologies that can overcome these challenges, improve detection accuracy, and reduce the associated risks of radiation, overdiagnosis, and overtreatment. A recent survey showed that some women in their 40s preferred to delay screening with mammography after being informed about the benefits and harms of the procedure [29]. Therefore, it is imperative to explore alternative technologies to mitigate these harms and enhance screening efficacy.

In the pursuit of improved screening technologies, one of the areas researchers have turned their attention to is microwave imaging (MWI). Unlike mammography, which uses ionizing radiation, MWI employs non-ionizing radiation, eliminating the associated risks of radiation exposure. This is especially important for younger women, who are more vulnerable to radiation-induced cancers due to their faster cell division rates. MWI works by measuring the dielectric properties of tissues, which vary significantly between healthy and cancerous tissue [30,31,32,33,34]. Cancerous tissues, characterized by higher water content and unique tissue decomposition and structure, exhibit elevated dielectric permittivity, while healthy tissues show lower permittivity values [35]. While edema also involves increased water content, its dielectric properties are influenced primarily by the distribution of extracellular fluid and lack the cellular irregularities and tissue decomposition associated with tumors. This distinction allows MWI to differentiate between edema and tumor tissues [36]. When microwaves interact with these tissues, the differences in water content cause distinct reflections, allowing MWI to detect even small tumors that might be missed by mammography, particularly in dense breast tissue, where overlapping densities often hinder accurate detection [34,37,38,39].

In addition to its superior sensitivity, MWI reduces false positives, minimizing the need for unnecessary biopsies and follow-up tests that can cause emotional distress, medical complications, and increased healthcare costs. This higher accuracy in detecting early-stage tumors and differentiating malignant from benign lesions makes MWI a reliable diagnostic tool, particularly in high-risk or dense breasts. The non-invasive nature of MWI, which requires no breast compression, also enhances patient comfort, further setting it apart from traditional mammography. Furthermore, MWI’s cost-effectiveness, portability, and potential for use in resource-limited settings make it an attractive option for broadening access to breast cancer screening. By leveraging scattering parameters to distinguish between different tissue types, MWI holds the potential to significantly improve breast cancer detection, offering a safer, more efficient, and patient-friendly alternative to mammography [33,34,37,38,39,40,41].

In addition to its advantages over mammography, microwave imaging (MWI) offers several key benefits over MRI. These include lower cost, greater portability, and the ability to avoid both ionizing radiation and the use of contrast agents, making MWI an especially attractive option for breast cancer screening in resource-limited settings. While MRI is highly effective for detecting soft tissue abnormalities, gadolinium-enhanced MRI may struggle to differentiate between benign and malignant lesions in dense breast tissue due to the complexities of signal interpretation. In contrast, MWI leverages microwave scattering, which is particularly sensitive to the unique dielectric properties of cancerous tissues, enabling it to more effectively detect tumors in dense breasts [33,34,38,39,42,43,44,45].

Many research groups have made significant strides in the development and testing of microwave breast imaging (MBI) prototypes. For instance, Micrima Ltd. (University of Bristol, Bristol, UK) conducted clinical trials using their MBI system, MARIA, involving 225 patients and achieving a sensitivity rate of 76% [46]. Similarly, Umbria Bioengineering Technologies (Rivotorto, Italy) tested their MammoWave prototype on 58 patients, reporting a sensitivity of 74% and employing Support Vector Machine (SVM)-based algorithms for automated lesion classification. The MammoWave system achieved an accuracy of 91%, a sensitivity of 84.4%, and a specificity of 97.2% [47]. Microwave Vision Group (MVG) in France also validated their Wavelia MBI prototype on 24 patients, demonstrating its ability to distinguish between benign and malignant lesions with an accuracy of 88.5% [37]. While these studies are promising, most of them have used cross-validation techniques, which tend to involve smaller datasets and may limit the generalizability of the results. Additionally, many of these studies have focused on simpler tasks, such as distinguishing between benign and malignant lesions or between malignant tissue and healthy tissue. However, they have not addressed the more complex task of differentiating malignant lesions (positive) from both benign lesions and healthy tissues (negative) in a unified framework, which is essential for accurate and reliable breast cancer detection.

At Mitos Medical Technologies (Istanbul, Turkey), we developed the SAFE (Scan and Find Early) device, a microwave-based imaging system designed to enhance breast cancer detection. The device underwent initial testing on a cohort of 54 subjects, achieving a sensitivity of 63% [39]. Building on this, our most recent study expanded the cohort to 113 patients. The focus of this study was to evaluate SAFE’s ability to differentiate benign and healthy tissues (negative) from malignant lesions (positive) [34]. This study achieved a sensitivity of 79% and specificity of 77% using 5-fold cross-validation. The primary objective was to assess SAFE’s capability to accurately classify malignant lesions from benign and healthy tissues, ultimately reducing unnecessary biopsies for patients already flagged by conventional screening methods like mammography.

This represents a significant advancement over previous efforts, primarily due to the increase in cohort size and methodological rigor [34,39]. In our first preliminary study, we used microwave images, but following that study, we decided to consistently use scattering parameters. These parameters represent the dielectric properties of tissues, which have been shown to differ significantly between cancerous and non-cancerous tissues, providing a more effective method for distinguishing between the two [39].

In contrast to earlier studies, which predominantly relied on cross-validation, we employed a more robust train–test split methodology. Specifically, the model was trained on data from 324 patients, validated on 112 patients, and tested on 15% (n = 80) of the unseen data. This approach provides a more reliable and independent evaluation of model performance, enhancing the generalizability of the results and reducing the risk of overfitting—an issue commonly associated with smaller training sets and cross-validation.

The aim of our study was to focus on a binary classification task, where we differentiate malignant lesions (positive) from both benign lesions and healthy tissues (negative). This is a more clinically relevant problem, as it combines benign and healthy tissues into a single negative class. This approach improves diagnostic accuracy, as it allows the device to focus specifically on identifying malignant lesions, while minimizing the confusion between benign and healthy tissues.

## 2. Materials and Methods

### 2.1. Patient Selection

The study received approval from the local ethics committee, and all protocols and procedures were conducted in accordance with institutional guidelines, ethical research standards, and the World Medical Association Declaration of Helsinki. Participants included patients aged 18 or older who visited the collaborating breast imaging clinic between May 2022 and October 2023 for screening, diagnostic assessment, or biopsy. Participation was entirely voluntary, with written informed consent obtained from each patient. Exclusion criteria included patients with a prior history of breast cancer and those with breast implants.

### 2.2. Device Description

**Figure 1:**
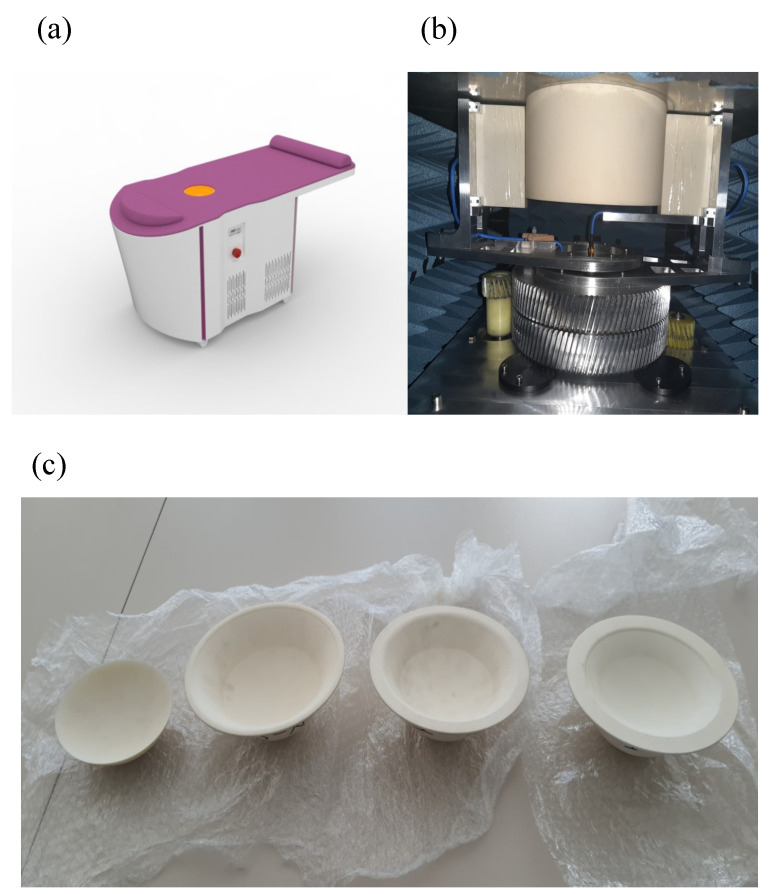
SAFE (Scan and Find Early) scanning system for clinical setup, system configuration and breast size cups. (**a**) The SAFE (Scan and Find Early) scanning bed system as used in a clinical setting, where data acquisition for each breast is completed in approximately 5 min; (**b**) the mechanically mobile bistatic system, where both transmitting and receiving antennas are positioned at 36 intervals of 10° each, generating 1296 measurements per frequency to ensure comprehensive data capture; and (**c**) patients are categorized according to breast size (1, 2, 3, and 4). Each patient is assigned a corresponding cup designed to fit their specific breast size for accurate measurements.

SAFE is a novel microwave breast screening device, developed by Mitos Medical Technologies. The device has two antennas, one designated for transmitting and the other for receiving microwave signals. Both antennas function within a ceramic medium for impedance matching, operating across a frequency range of 0.6–8 GHz with a sampling interval of 200 MHz. These antennas are connected to a 2-port vector network analyzer (P9372A Keysight USB) and are housed within a cubical enclosure lined internally with microwave absorbers. The enclosure features a ceramic cylinder embedded in a cavity, designed to accommodate the patient’s breast in a prone position. Adjustable ceramic cups are provided as part of the cylindrical setup to account for variations in breast size. The antennas rotate azimuthally, capturing signals in a configuration where each transmitting position corresponds to multiple receiving positions. In this arrangement, both the transmitting and receiving antennas have 36 positions, with each position separated by a 10° interval. The SAFE system completes data acquisition in approximately 5 min per breast. The 10° interval captures diverse angles, enhancing measurement variety and enabling accurate differentiation between tissue types (e.g., malignant vs. benign) using S11 and S21 parameters (Figure 1).

### 2.3. Data Categorization

**Figure 2:**
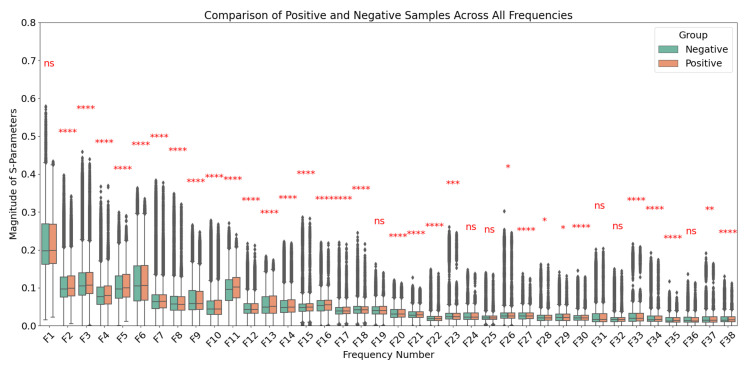
Comparison of S-parameters between positive (malignant) and negative (benign or no pathology) groups across frequencies ranging from 0.6 GHz to 8 GHz in 0.2 GHz increments. Welch’s *t*-test was applied at each frequency to assess statistical significance, with *p*-value thresholds represented as follows: *p* < 0.05 (*), *p* < 0.01 (**), *p* < 0.001 (***), and *p* < 0.0001 (****), with “ns” denoting not significant (*p* > 0.05). Data are presented as mean ± standard deviation for each group. Significant differences at specific frequencies highlight the distinct characteristics between the two groups.

Patients with BI-RADS mammogram readings of categories 1 and 2, as well as those who underwent biopsy with benign pathology results, were categorized as the negative group. Patients with malignant pathology findings confirmed by biopsy were categorized as the positive group. To ensure this categorization reflected meaningful distinctions, Welch’s *t*-test was applied to compare the two groups for each frequency individually (Figure 2) and for the combined frequencies (Supplementary Figure S1a). A *p*-value threshold of 0.05 was used to determine statistical significance. The significance levels are indicated in the figures as follows: a single asterisk (*) for *p*-values between 0.01 and 0.05, two asterisks (**) for *p*-values between 0.001 and 0.01, three asterisks (***) for *p*-values between 0.0001 and 0.001, and four asterisks (****) for *p*-values less than 0.0001. This statistical framework provides robust support for the classification of patients with malignant pathology as positive and those with benign or no pathology as negative.

Patients were also categorized into subgroups based on factors such as age, breast density, and breast size to check how the model performance alters across those groups. Breast density was recorded for those who underwent mammography or MRI before or after biopsy, while it remained unknown for patients examined solely by ultrasound. Density was assessed using the BI-RADS scale, as follows: A—predominantly fatty; B—scattered fibroglandular tissue; C—heterogeneously dense; and D—extremely dense [48]. Given the limited sample size, patients were divided into two density categories: non-dense (BI-RADS A and B) and dense (BI-RADS C and D). Age was divided into two groups: younger (18–47 years) and older (48 years and above), with the median age of participants as the cutoff. Breast size was also classified into two groups based on the ceramic cups used to support the breast during scanning: small (cup sizes 1 and 2) and large (cup sizes 3 and 4), with cup selection determined by the medical technician performing the scan. Breast lesions were categorized into three groups based on size: T1, for lesions measuring between 0 mm and 20 mm; T2, for lesions between 21 mm and 50 mm; and T3, for lesions larger than 50 mm. Due to the limitations of the dataset, T4 lesions, which had spread to adjacent tissues and were larger than 50 mm in size, were not included in the analysis.

### 2.4. Data Analysis

To enable automated breast lesion classification, both S11 and S21 parameters—proportional to the electromagnetic field emerging after the incident wave radiated by the transmitting antenna has interacted with breast tissue—were collected for each negative group (healthy, benign) and positive group (malignant) at 38 different frequency points. The measurements were conducted using vector network analysis (VNA), where the intermediate frequency (IF) bandwidth was adjusted to ensure a low noise ratio. The raw S11 and S21 data, denoised by the VNA, were directly used in their original time-series form without applying additional transformations.

For each patient sample, a 36 × 36 matrix was constructed, where each row represented the magnitude of S11 and S21 measurements across 36 receiver positions. The total number of S-parameters equaled 36 × 36 due to the 36 transmitter positions. This process was repeated for 38 distinct frequencies spanning the range of 0.6–8 GHz. Principal component analysis (PCA) was applied to the 36 × 36 matrices at each frequency. The dimensionality of the data was reduced by summing the singular values obtained from the decomposed vectors. As a result, each patient was represented by 38 features, corresponding to the 38 frequency points. The features for all patients were compiled into a feature matrix.

To classify the negative and positive samples, the extreme gradient boosting (XGBoost) algorithm—a tree-based ensemble method—was utilized due to its ability to handle structured datasets, its resilience to overfitting through built-in regularization, and its effectiveness in processing imbalanced datasets. The algorithm was implemented using the Python programming language and the XGBoost library (xgboost v2.0.2). Histopathological results served as the ground truth for training and validation. XGBoost minimizes residuals and enhances predictive performance by combining multiple weak learner base estimators [49]. Prediction probabilities were calculated to determine malignancy, with a default threshold value of 0.5 used to classify cases as malignant. Patients with probabilities exceeding this threshold were classified as likely to have malignant breast cancer.

Previously, it was observed that breast size significantly impacts model performance [39]. Therefore, to prepare the dataset for training, samples were stratified by breast size to ensure balanced representation: 2× healthy samples, 1× benign sample, and 1× malignant sample from each breast size were selected. This strategy was designed to better reflect real-world clinical scenarios, where healthy cases are more prevalent. In total, the dataset comprised 80 breasts, of which 59 were negative (healthy + benign) and 21 were positive (malignant). The full dataset consisted of 526 samples, with a test set designed to include 15% of the total samples, adjusted to reflect the distribution observed in clinical practice. For training and validation, with the remaining 446 samples, we applied stratified splitting based on labels (negative/positive) and breast sizes using a 3:1 split ratio. The final split included 168 negative samples and 166 positive samples for the training set, and 59 negative samples and 53 positive samples for the validation set. Sensitivity, specificity, and accuracy metrics were calculated for each group, and the results were combined to evaluate the overall performance of the models in detecting malignant breast cancer cases. In addition to evaluating the overall performance of SAFE in classifying positive and negative samples, the performance of SAFE within specific subgroups was analyzed, focusing on breast density, breast size, and age groups. These subgroup analyses provided insights into how the model performed across varying sample characteristics. All results are presented using ROC curves, demonstrating the model’s performance across different thresholds for both overall classification and subgroup-specific analyses.

To compare the negative and positive groups across age categories, Welch’s *t*-test was applied to assess the differences in age both within the groups (negative vs. positive) and between the groups (young vs. older patients). Similarly, to evaluate the distribution of the negative and positive groups across different breast density and breast size categories, the chi-square test was utilized. Lastly, to determine whether lesion size differed between the training and test sets, Welch’s *t*-test was performed. All statistical analyses were performed using the Python library statannot v.0.2.3. For the significance levels, *p*-values were categorized as follows: a *p*-value less than 0.0001 was considered highly significant (****), *p*-values between 0.0001 and 0.001 indicated strong significance (***), *p*-values ranging from 0.001 to 0.01 were categorized as moderately significant (**), and *p*-values between 0.01 and 0.05 represented mild significance (*).

## 3. Results

In this study, a total of 526 patients were included, with 286 classified as the negative group (healthy patients and benign patients) and 240, diagnosed with malignant breast cancer, classified as the positive group. The patients’ ages ranged from 19 to 74 years, with a mean age of 47.2 ± 9.7 years. A slight but statistically significant difference was observed between the negative and positive samples in the older age group (*p*-value between 0.05 and 0.01) (Supplementary Figure S1b). No significant differences were found when comparing the negative and positive samples within the breast density and breast size groups, nor were there statistically significant differences in the distribution of patients across these categories (Supplementary Figure S1c,d). Additionally, a significant difference was observed between the younger and older patient groups, confirming that the age threshold of 48 years effectively separated the two categories (Supplementary Figure S1b). Among the malignant cases, lesion sizes ranged from a minimum of 8 mm to a maximum of 46 mm, with an average size of 22.3 ± 9.2 mm.

Notably, the negative groups consisted of more patients than the positive group in both small breasts (202 negative, 174 positive) and large breasts (84 negative, 66 positive). After splitting the data into training, validation, and test sets, the total numbers were 334 for the training set, 112 for the validation set, and 80 for the test set. Of the 80 test samples, 21 were positive samples, and 59 were negative samples. Within the test set, 47 out of 80 breasts were categorized as non-dense (BI-RADS types A and B), while 23 were classified as dense (BI-RADS types C and D). Density measurements were unavailable for the remaining 10 breast samples due to the exclusive availability of ultrasonography (US) images. In the smaller-breast subgroup of the test data, the count of dense breasts (n = 20) closely paralleled that of non-dense breasts (n = 28). Conversely, in the larger-breast subgroup, the number of dense breasts (n = 3) was notably lower than that of non-dense breasts (n = 19). Additionally, the test dataset included 42 patients categorized as old (Age ≥ 48) and 38 patients categorized as young (Age < 48) (Supplementary Table S2).

Upon reviewing the histopathological findings of the lesions, 286 out of 491 lesions formed by positive and negative samples (malignant and benign lesions) were confirmed with specific histological diagnoses. The most common finding was invasive ductal carcinoma (IDC), which accounted for 82 cases (approximately 28.7%) of the total positive samples. Additionally, fibroepithelial lesions, fibrosis, and fibroadenomas were observed in 68 cases (approximately 23.8%). Other notable findings included chronic inflammation (n = 10), with additional histological diagnoses each occurring in fewer than 10 cases. These predominant histological findings were more common in the positive samples compared to the negative samples, which showed a less varied distribution of findings. In the test set, 21 positive (malignant) and 21 negative (benign) samples were analyzed for histological distribution. Of these, 14 samples were diagnosed with invasive ductal carcinoma (IDC), and 10 samples had either fibroepithelial lesions or fibrosis. The remaining cases included invasive apocrine carcinoma and metastatic carcinoma and chronic inflammation (Supplementary Figure S2a, Supplementary Table S1).

**Figure 3:**
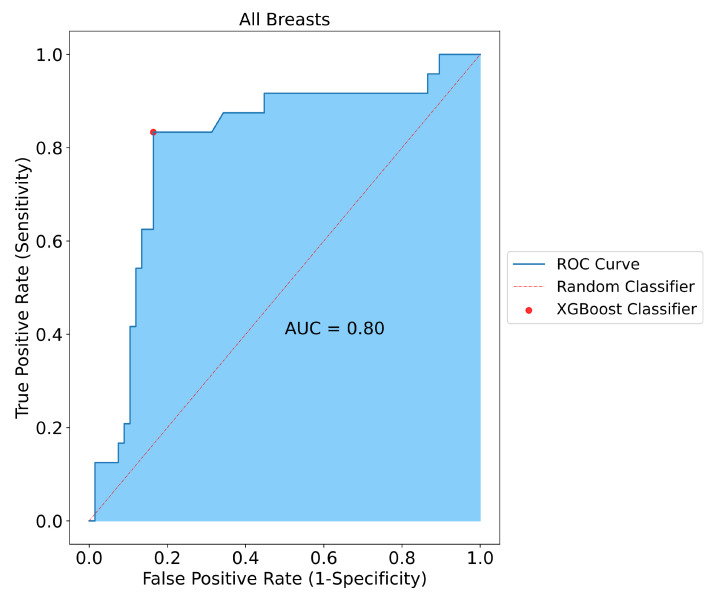
Classification results for all breasts included in the study. SAFE correctly classified 65 out of 80 tested subjects with 0.80, 0.81, and 0.81 for sensitivity, specificity, and accuracy, respectively.

Following the classification of positive and negative samples, the model performance for all breasts was measured. SAFE was able to correctly classify 65 of the samples out of 80 patients in the test group (false positive: 11, false negative: 4). As a result, the sensitivity, specificity, and accuracy of the model performance for all breasts were measured as 80%, 81%, and 81%, respectively (Figure 3).

**Figure 4:**
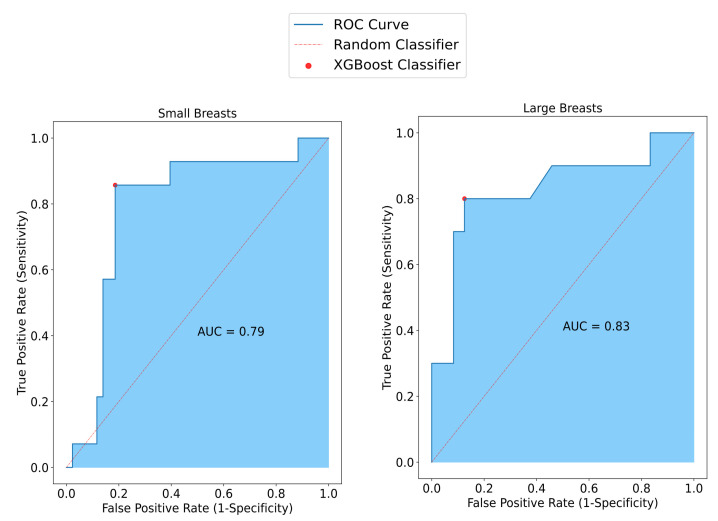
SAFE classification results vary with breast size. In smaller breasts, sensitivity reaches 81%, surpassing the 71% observed in larger breasts. Conversely, specificity decreases to 81% in larger breasts compared to 86% in smaller ones. Accuracy improves from 82% in larger breasts to 78% in smaller ones.

To assess performance variations relative to breast size, the model’s efficacy was evaluated separately for large and small breasts. Notably, in small breasts, SAFE demonstrated an accuracy of 82%, while in large breasts, it achieved a lower accuracy rate of 78%. Moreover, the model applied to small breasts exhibited a superior specificity of 86% compared to the 81% specificity observed in large breasts. Correspondingly, the model’s sensitivity in small breasts was measured at 81%, whereas in large breasts, it notably dropped to 71% (Figure 4).

**Figure 5:**
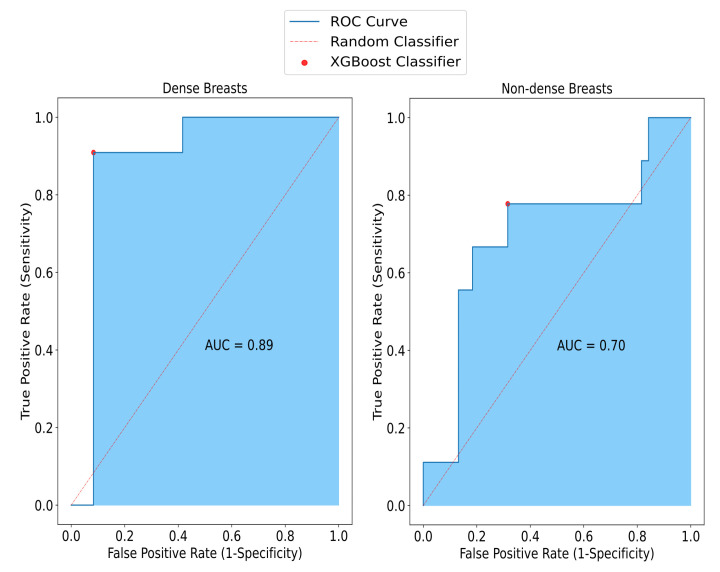
The impact of density on SAFE classification results. Sensitivity, specificity, and accuracy rates obtained from non-dense small breasts are 67%, 82%, and 79%, respectively, whereas in dense breasts, these results are 91%, 92%, and 91%, respectively.

Considering the performance of the overall model in terms of breast density, in non-dense breasts, SAFE achieved an accuracy of 79%, while it was measured as 91% in dense breasts. Also, the model had higher specificity (92%) for dense breasts than the model for the non-dense breasts (82%). Similarly, the sensitivity of the model for the non-dense breasts (67%) was lower than for the dense breasts (91%). These performance results only considered the breast samples that had information on breast density (Figure 5).

**Figure 6:**
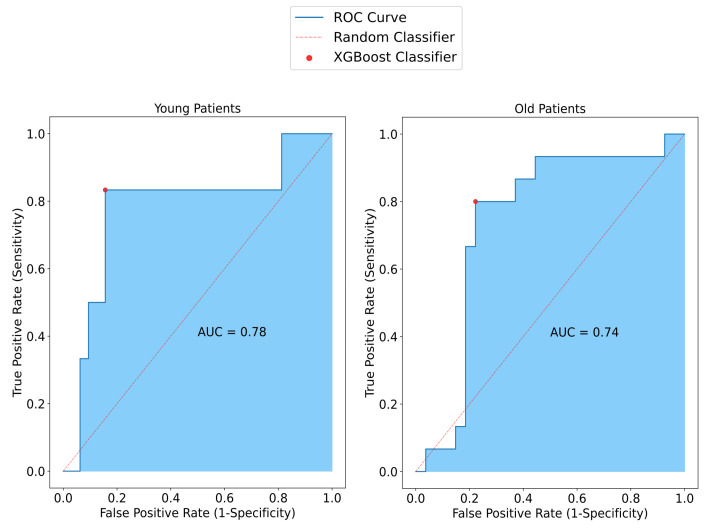
Representation of SAFE performance in relation to age among patients with small breasts. In the younger group, sensitivity, specificity, and accuracy were measured at 83%, 84%, and 84%, respectively. Conversely, in the older patient group, higher values were observed compared to the younger group, with sensitivity, specificity, and accuracy measured at 80%, 78%, and 79%, respectively.

To investigate the effect of the patient’s age on the model, the model performance for both the old age group and the young age group was assessed. The sensitivity of SAFE was lower in the old patient group (80%) than in the young patient group (83%). Similarly, comparing the specificity of the patients, the specificity of SAFE for older patients (78%) was also lower than for the younger patients (84%). Lastly, the accuracy results for old and young patients were 79% and 84%, respectively (Figure 6).

The model’s performance in classifying histological findings was evaluated on the test set. For invasive ductal carcinoma (IDC), the model accurately classified 11 out of 14 cases (78.6%) of this malignant lesion. For benign lesions, such as fibroepithelial lesions and fibrosis, the model demonstrated excellent performance, correctly classifying 90% of all cases. Due to the limited number of other histological findings in the test set, their classification performance was not considered. Finally, the distribution of lesion sizes between the training and test sets was examined. Statistical analysis using Welch’s *t*-test revealed no significant difference in lesion size between the two groups (Welch’s *t*-test, *p* = 0.05), indicating that the dataset is balanced with respect to lesion size (Supplementary Figure S2b).

**Figure 7:**
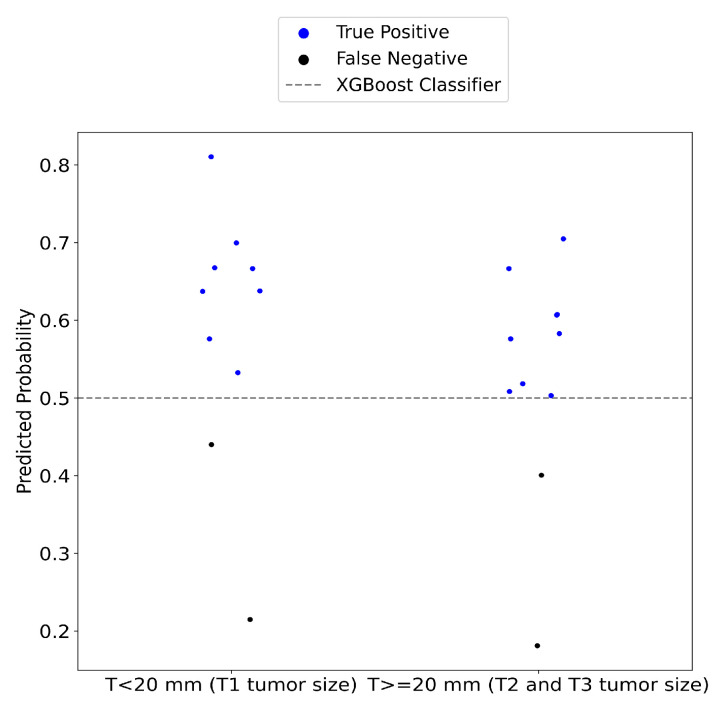
The performance of the SAFE system was evaluated across different cancerous lesion groups. In group 1, consisting of T1 tumor lesions, the system correctly identified 80% of the malignant tumors. For group 2, which includes T2 and T3, the sensitivity of the SAFE system was measured at 82%.

The performance of the device was also assessed in terms of lesion size. SAFE successfully identified 8 out of 10 T1 lesions (smaller than 2 cm) and accurately detected 9 out of 11 larger T2 and T3 lesions (Figure 7).

## 4. Discussion

The aim of our study was to focus on a binary classification task to differentiate malignant breast lesions (positive) from benign lesions and healthy tissues (negative). This approach is clinically significant because it combines both benign and healthy tissues into a single negative category, which more closely aligns with the diagnostic challenge in clinical settings. By adopting this binary classification framework, the device is able to focus on accurately identifying malignant lesions while minimizing the risk of misclassifying benign tissues. This approach improves diagnostic accuracy, helping to reduce the confusion between benign and healthy tissues, which is crucial for effective clinical decision making.

To achieve this classification, we utilized the SAFE microwave breast imaging device to gather data from patients with both malignant and non-cancerous breast tissues (comprising healthy and benign patients). The absolute values of the S11 and S21 complex matrices, representing the electromagnetic properties of the breast tissue, were used as feature variables in the predictive modeling. We applied the XGBoost machine learning algorithm, known for its robustness in handling complex, high-dimensional data, to classify non-cancerous individuals and malignant breast cancer patients [49].

In our analysis, we compared the negative and positive samples within each age group. A slight but statistically significant difference was observed in the older age group, with a *p*-value falling between 0.05 and 0.01. This indicates that the negative and positive samples were well-matched across age groups, ensuring a balanced representation of both healthy and unhealthy patients within similar age ranges. The balanced matching of samples is essential for the model’s performance, as it strengthens the model’s robustness by providing a consistent and diverse dataset for training. This approach ultimately leads to more reliable and generalizable predictions.

Further, when comparing the negative and positive samples within each breast density and breast size group, no significant differences were found, which suggests that the dataset maintains a balanced representation across both sample types within these categories. Similarly, when comparing the number of patients in each density and breast size group, no statistically significant differences were observed. This reinforces the well-distributed nature of the selected samples, which is crucial for effective model training.

In contrast, when comparing the younger and older patient groups, a significant difference was observed between the two age categories, indicating that the age threshold of 48 years effectively separates the groups. This finding confirms the appropriateness of the chosen age cutoff, ensuring a clear distinction between the two age categories for the further performance analysis of the SAFE system based on age.

Notably, in this study, the dataset consisted of T1 and T2 lesions, with only a very small number of T3 lesions (n = 1) across the training, validation, and test sets. This structure was intentionally designed to focus on small breasts, particularly in the test set, allowing for a more targeted evaluation of the SAFE model’s performance on this subset. The overall number of T3 lesions was very low, accounting for only 23 lesions out of 491 lesions (240 malignant lesions and 251 lesions samples). Additionally, the number of small-breast samples was higher than that of large breasts, further emphasizing the focus on small-breast categories.

In our first preliminary study, we employed qualitative image algorithms for the detection of breast cancer patients, reconstructing the MWI images by the localization of breast cancer. We scanned a total of 115 patient breasts and had a sensitivity of 63% in the overall patient samples and concluded that breast size dramatically affects results, where the device had 51% sensitivity in smaller breasts, while in larger breasts it had a sensitivity of 74% [39].

In the current study, the performance of SAFE was evaluated across both small and large breasts, and the performance of the device was remarkably similar between the two groups. However, a slight advantage was observed in smaller breasts, which may be attributed to the sample size of the large-breast group. Smaller breasts may allow for more direct coupling between antennas, potentially reducing the interaction of electromagnetic waves with the breast tissue. In scenarios where antennas are closely coupled, detecting subtle variations in the electromagnetic field becomes particularly challenging [39]. This phenomenon could potentially affect SAFE’s ability to detect breast cancer in patients with smaller breasts. However, the noise removal process implemented during preprocessing appeared to mitigate these challenges, leading to improved performance of SAFE in smaller breasts. Despite the differences in model performance between breast sizes, preliminary results indicate notable improvements in SAFE’s ability to detect breast cancer in smaller breasts.

Breast density can significantly influence the performance of mammography. Mammography typically demonstrates higher sensitivity in non-dense breasts (75%), which decreases to 51.3% in dense breasts [39]. In our preliminary findings, we demonstrated that SAFE exhibits superior performance regardless of breast density [39]. In our current expanded study, sensitivity rates of 91% for dense breasts and 67% for non-dense breasts were observed. Notably, dense breasts were evenly distributed across both the large- and small-breast test sets. However, non-dense breasts were underrepresented, with only three samples in the large-breast test set and nine in the positive group test set out of 21 samples. SAFE accurately classified all three non-dense breasts within this subset. Given the impact of breast size on model performance, we plan to increase the number of non-dense breast samples, particularly within the large-breast subset, to further enhance the sensitivity beyond the current 67%. Until this time, the high performance in dense breasts is promising as a potential supplemental screening tool in dense breasts.

In mammography, screening is recommended for older patients due to the cumulative harmful effects of ionizing radiation [6,7,8,9]. However, SAFE, which operates using non-ionizing low electromagnetic frequencies, poses no such risks and achieves 83% sensitivity in younger patients. However, the cohort size for malignant patients in the younger age group (n = 6) was small, and increasing this sample size would allow for more precise predictions. The results for younger patients were consistent with SAFE’s previous performance in our preliminary study [39]. In the older patient cohort, SAFE demonstrated enhanced performance, with sensitivity, specificity, and accuracy rates of 80%, 78%, and 79%, respectively.

The prognosis of breast cancer is greatly influenced by lesion size. Early diagnosis is crucial to prevent metastases and increase survival [50,51]. SAFE successfully identified 80% of T1 (smaller than 2 cm) malignant lesions. Additionally, SAFE demonstrated an 82% detection rate for larger lesions exceeding 2 cm in size. These results suggest that SAFE could be a promising tool for screening, capable of detecting cancerous tissue before significant progression occurs.

To evaluate the generalizability of our model and check for signs of overfitting, we also examined the training and validation loss curves. A typical indication of overfitting would be a scenario where the training loss continues to decrease while the validation loss increases over time. In contrast, our model demonstrated a consistent decrease in both training and validation loss throughout the training process, as shown in Supplementary Figure S3. This indicates that the model generalizes well to unseen data and has not overfitted to the training set, which is a key strength of the model’s robustness.

In comparing our study’s binary classification framework with previous microwave breast imaging (MBI) research, several key distinctions and advancements emerge that highlight the significance of our work. Our study addresses a binary classification task that differentiates malignant lesions from both benign and healthy tissues, which not only aligns more closely with real-world diagnostic needs but also reduces confusion in clinical decision making. By grouping benign and healthy tissues together as a single negative category, our framework minimizes unnecessary complexity and allows clinicians to focus more precisely on the critical task of identifying malignant lesions. This approach is especially important in clinical settings where the primary concern is detecting cancer, and reducing the number of false positives can lead to fewer follow-up procedures.

One of the novel contributions of our study is the ability of SAFE to effectively classify negative samples (both benign and healthy tissue) from positive samples (malignant tissue). This binary classification framework has broad implications for early detection and pre-screening. Since SAFE distinguishes malignancies from healthy tissue early in the process, it can be utilized prior to traditional screening technologies like mammography or ultrasound. This is a crucial advancement, particularly for dense breasts, where traditional methods often fall short. By identifying suspicious lesions early, SAFE provides a first line of defense in breast cancer detection, potentially reducing the need for more invasive diagnostic procedures.

Furthermore, SAFE’s ability to distinguish benign from malignant lesions helps facilitate the biopsy process. In current clinical practice, biopsies are often used to confirm the presence of cancer after initial imaging has identified suspicious areas [52]. SAFE’s pre-screening capability can help refine this process by more accurately classifying malignant lesions from benign ones, ensuring that biopsies are performed on truly suspicious lesions, and reducing the number of unnecessary biopsies for benign cases. This could lead to fewer procedures, reduced patient anxiety, and more efficient use of medical resources.

There are many limitations in our study: one limitation is that while the cohort size is larger compared to previous preliminary studies [34,39], the choice to use a holdout validation method introduces certain limitations. Specifically, using 20% of the dataset as a test set limits the data available for model training. Even though we applied a stratified split to the data, this can potentially affect the model’s ability to learn from the full range of patient data, especially if the test set contains rare or outlier cases that are underrepresented in the training set.

Intriguingly, although the model demonstrated good performance in classifying benign lesions, the classification of IDC was slightly more challenging. The lower accuracy for IDC (78.6%) may be attributed to several factors. First, IDC tumors are often heterogeneous, with varying tissue structures, cellularity, and vascularization across different regions of the tumor. This heterogeneity can lead to variations in the microwave signal patterns, making it harder for the model to consistently identify IDC samples [53].

Furthermore, the water content in IDC tumors could contribute to these challenges. Malignant tumors, particularly IDC, often have a different water content distribution compared to benign lesions. The presence of necrotic regions and increased blood supply in IDC could alter the water retention properties of the tissue, influencing the microwave signal scattering in ways that are less predictable compared to the more uniform patterns observed in benign lesions [54].

Future work could focus on enhancing the model’s ability to discriminate between different tumor types by incorporating data from a wider variety of malignant subtypes. This would not only improve the model’s robustness in identifying IDC but also help in better classifying other rare malignant tumors that were underrepresented in this study. Additionally, incorporating features related to water content and tissue composition could provide valuable insights into how these factors influence microwave signal interactions, potentially leading to improved classification accuracy for both malignant and benign lesions.

A significant limitation in this study arises from the subjective assessment of breast cup size. Since breast cup size is typically assessed based on visual or physical measurement, it is prone to variability between healthcare providers. This subjectivity introduces a potential source of mismatch error, particularly in categorizing patients into large- or small-breast size groups. Inconsistent categorization may affect the study’s analysis, as breast size was found to influence the performance of the SAFE device. For instance, if a patient’s breast size is categorized incorrectly, it could impact the model’s sensitivity and specificity for different patient subgroups, leading to inaccurate conclusions. To address this, future studies could implement a more standardized or objective system for breast size classification, such as using specific anatomical measurements or imaging-based techniques, to minimize variability and ensure consistency across patients.

Another limitation of this study involves the missing breast density categorization in 10 test samples. These patients underwent ultrasound (US) examination rather than traditional mammography, which led to the absence of breast density data. Breast density is a critical factor in the performance of many breast cancer detection techniques, including SAFE. The lack of density categorization in a portion of the test dataset limits the ability to evaluate how SAFE performs across different breast densities. These missing data could introduce a bias in the study, as the device’s sensitivity might vary depending on whether the breast tissue is dense or non-dense. To improve the accuracy of future studies, it is important to ensure that all test samples are categorized for breast density, either through mammography or alternative techniques such as ultrasound or MRI. This would provide a more comprehensive analysis of the device’s performance across various tissue types and densities.

Although the current study offers valuable insights into the performance of the SAFE device, its generalizability to broader populations is limited. The cohort may have been drawn from a specific geographic region or demographic group, which may not fully represent the global population. For example, the sample might be biased towards particular ethnicities or risk groups, which could affect the model’s performance in other populations. Differences in breast tissue characteristics, cancer prevalence, and genetic factors could influence the device’s efficacy. To enhance generalizability, future studies should include a more diverse cohort from different geographical locations, ethnic backgrounds, and risk categories. A multi-center, international study could provide a broader evaluation of the device’s performance and ensure that the findings are applicable to a wider range of patients.

## 5. Conclusions

The SAFE microwave breast imaging system demonstrates significant promise in the binary classification of malignant lesions versus benign and healthy tissues, aligning closely with real-world clinical needs. By combining benign and healthy tissues into a single negative category, the system simplifies the diagnostic process and reduces confusion, improving overall diagnostic accuracy. The system’s ability to identify malignant lesions early, especially in dense breasts, offers a valuable tool in pre-screening and early detection, which can complement traditional screening methods like mammography.

In addition, SAFE’s capacity to differentiate benign from malignant lesions helps in improving the biopsy process, reducing unnecessary procedures and associated patient anxiety. While SAFE performs well across varying breast sizes and densities, further enhancements in breast size classification and breast density categorization are needed to improve performance consistency. Larger and more diverse cohorts would also help enhance the generalizability of the system.

Overall, SAFE provides a non-invasive, non-ionizing approach to breast cancer detection with potential applications in early diagnosis, screening, and biopsy guidance, representing an important step forward in breast cancer care.

## Data Availability

The data are not publicly available due to Governmental and Institutional (Marmara University Hospital Breast Center) regulations and non-violation of patients privacy.

## Supplementary Materials

The following supporting information can be downloaded at: https://www.mdpi.com/article/10.3390/cancers17020214/s1, Figure S1. Statistical Comparisons of patient groups by age, breast density, and breast Size, including raw data analysis between patient groups. (a) Comparison of negative and positive patient groups for all frequencies combined. The **** symbol indicates that the *p*-value is less than 0.0001. (b) Comparison of negative and positive patient groups within older and younger age groups. The **** symbol indicates that the *p*-value for the comparison between older and younger groups, for both negative and positive patients, is less than 0.0001. A slight but statistically significant difference was observed between negative and positive groups within the older age group (*, *p*-value between 0.05 and 0.1), while no statistical difference was observed in the younger age group. (c,d) No significant differences were observed across or between groups in terms of breast size or breast density. For breast size, comparisons were made between small and large breasts for each patient group, as well as between negative and positive groups within each breast size. For breast density, comparisons were made between non-dense and dense breasts for each patient group, as well as between negative and positive groups within each density group. Welch’s *t*-test was applied for the comparison of raw data and age groups, while the Chi-square test was used for comparisons of breast size and density categories. Figure S2. Histopathological findings for all groups of patients and lesion size comparisons between train and test groups. (a) The top 8 histopathological findings of lesions from positive and negative groups. A total of 286 confirmed histopathological results were analyzed. Among these, 82 cases (28.7%) were identified as invasive ductal carcinoma, while 23.8% were classified as fibroepithelial lesions, fibrosis, and fibroadenomas. Comparison of lesion sizes between the training and test groups. No significant differences were observed (ns). Welch’s *t*-test was applied, with a *p*-value threshold of 0.5. Figure S3. Training and Validation Loss During Model Training. The plot illustrates the training and validation loss during model training. The x-axis represents the number of iterations through the entire training dataset, while the y-axis represents the log loss. As the training loss decreases, the validation loss also decreases, indicating consistent model performance. Table S1. Comprehensive List of Histological Findings for Lesions Across All Patient Groups. The table provides a detailed summary of all histological findings for lesions across the patient groups, along with their respective counts. A total of 286 histological findings were documented. Table S2. Distribution of Patient Samples Across Various Categories. This table provides the distribution of patient samples across different categories, including the number of healthy and unhealthy samples, as well as the breakdown of training, validation, and test samples. Additionally, it presents the categorization of test samples based on breast density (dense vs. non-dense) and age (young vs. old). All sample sizes were shown in relation to breast size.
