# Supplementary material for: XGBoost Enhances the Performance of SAFE: A Novel Microwave Imaging System for Early Detection of Malignant Breast Cancer
Source: Cancers (Basel). 2025 Jan 10;17(2):214. doi: 10.3390/cancers17020214 (PMC11764354; doi:10.3390/cancers17020214)

a)

Histopathological Finding	Number of Findings
invasive ductal carcinoma	82
fibroepithelial lesion	30
fibrosis	20
fibroadenoma	18
chronic inflammation	10
invasive lobular carcinoma	8
metastatic lymph node	6
ductal carcinoma in situ	6

b)

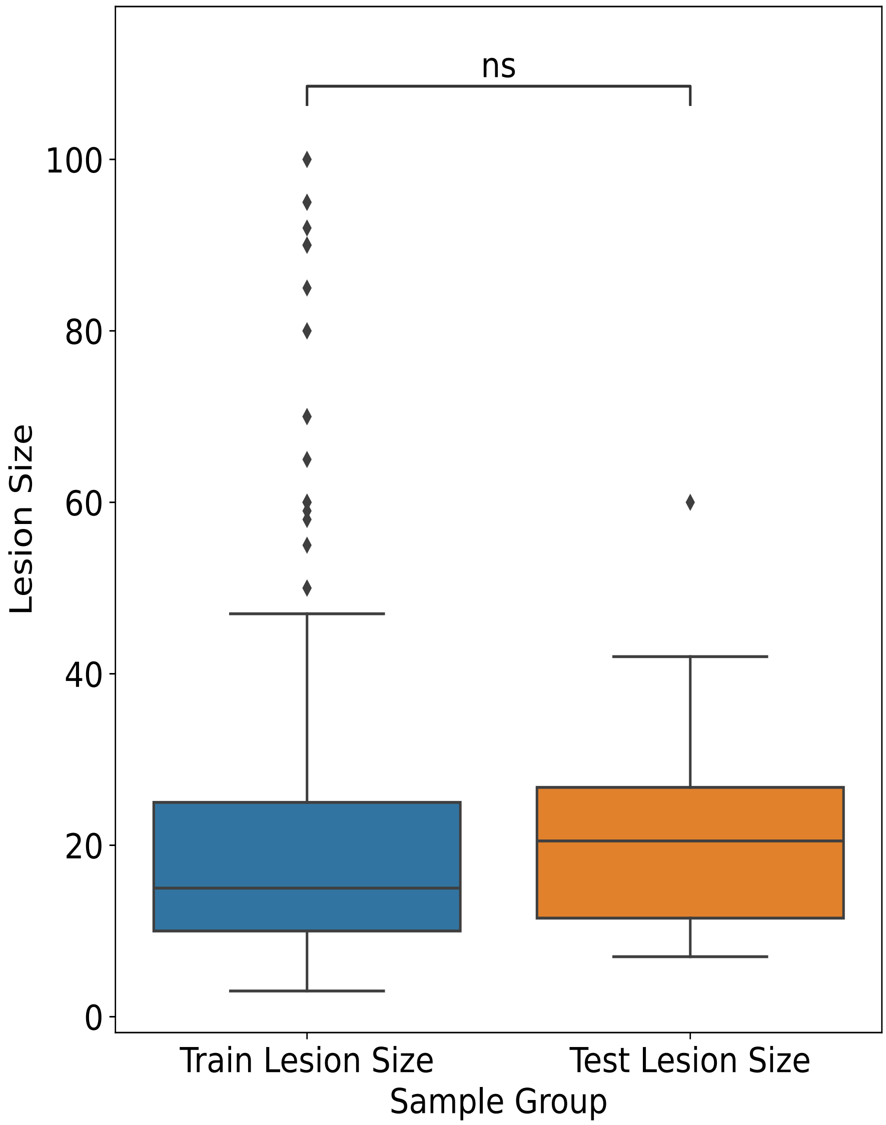

Supplement: Supplementary file 1 [file cancers-17-00214-s001.zip › Supplementary_Figure2.pdf]
