# Supplementary figures and images for: XGBoost Enhances the Performance of SAFE: A Novel Microwave Imaging System for Early Detection of Malignant Breast Cancer
Source: Cancers (Basel). 2025 Jan 10;17(2):214. doi: 10.3390/cancers17020214 (PMC11764354; doi:10.3390/cancers17020214)

a)

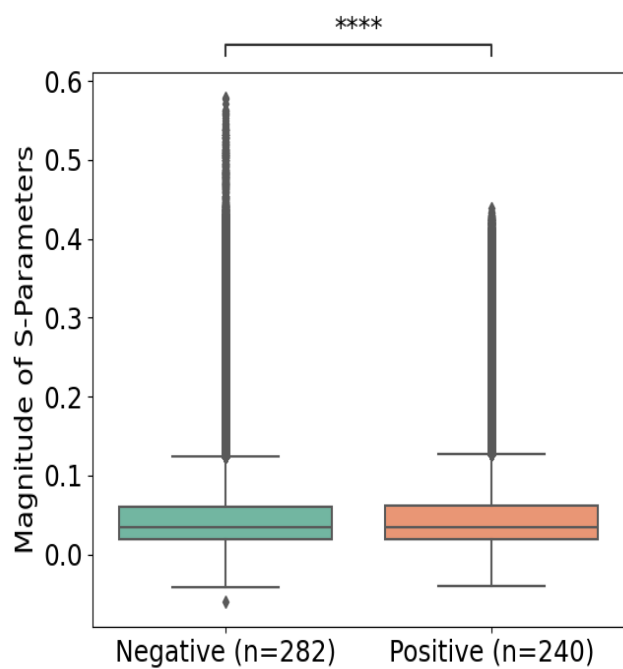

b)

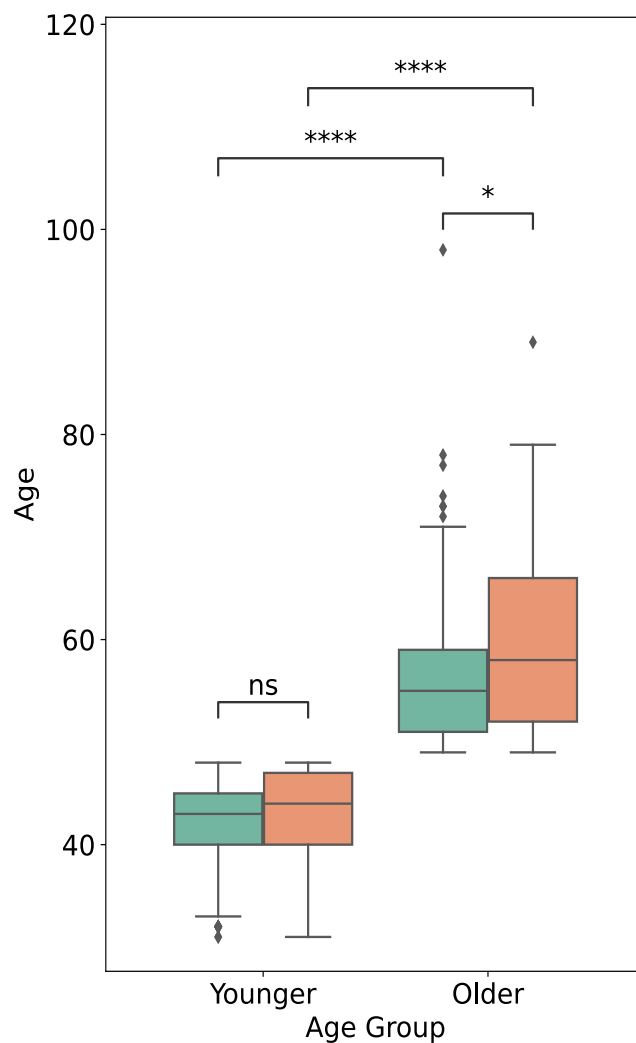

c)

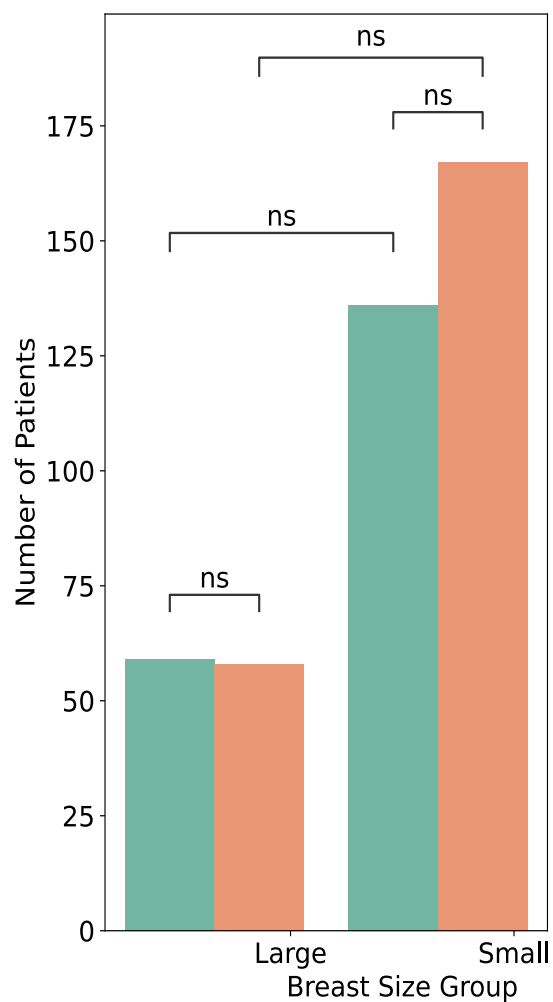

d)

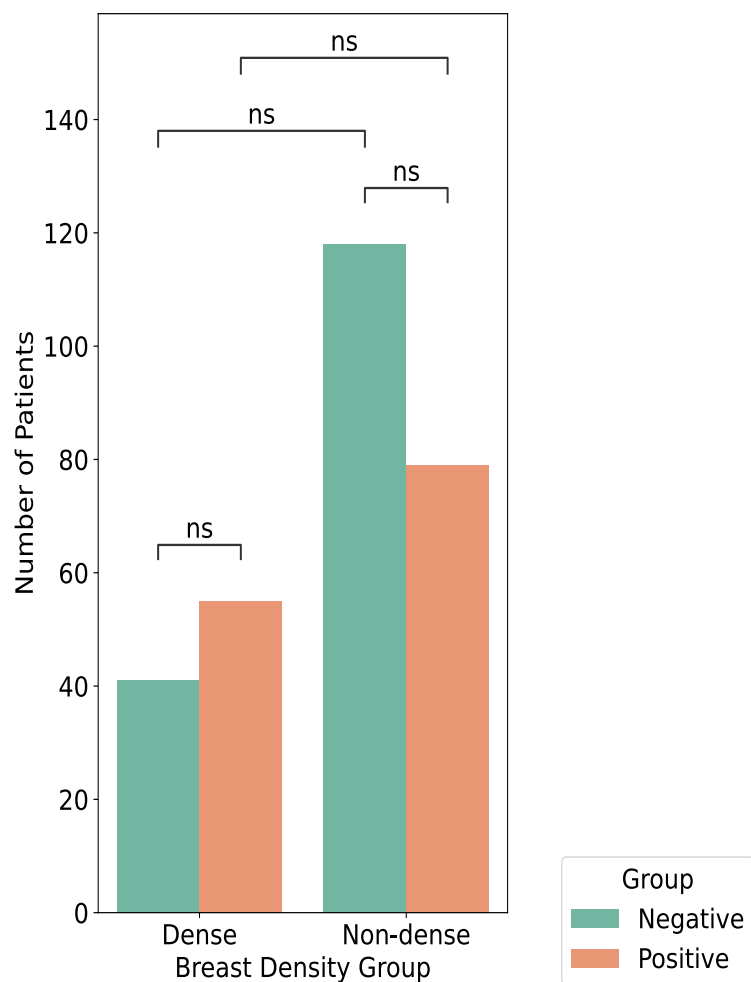

Supplement: Supplementary file 1 [file cancers-17-00214-s001.zip › Supplementary_Figure1.pdf]

Train vs Validation Loss

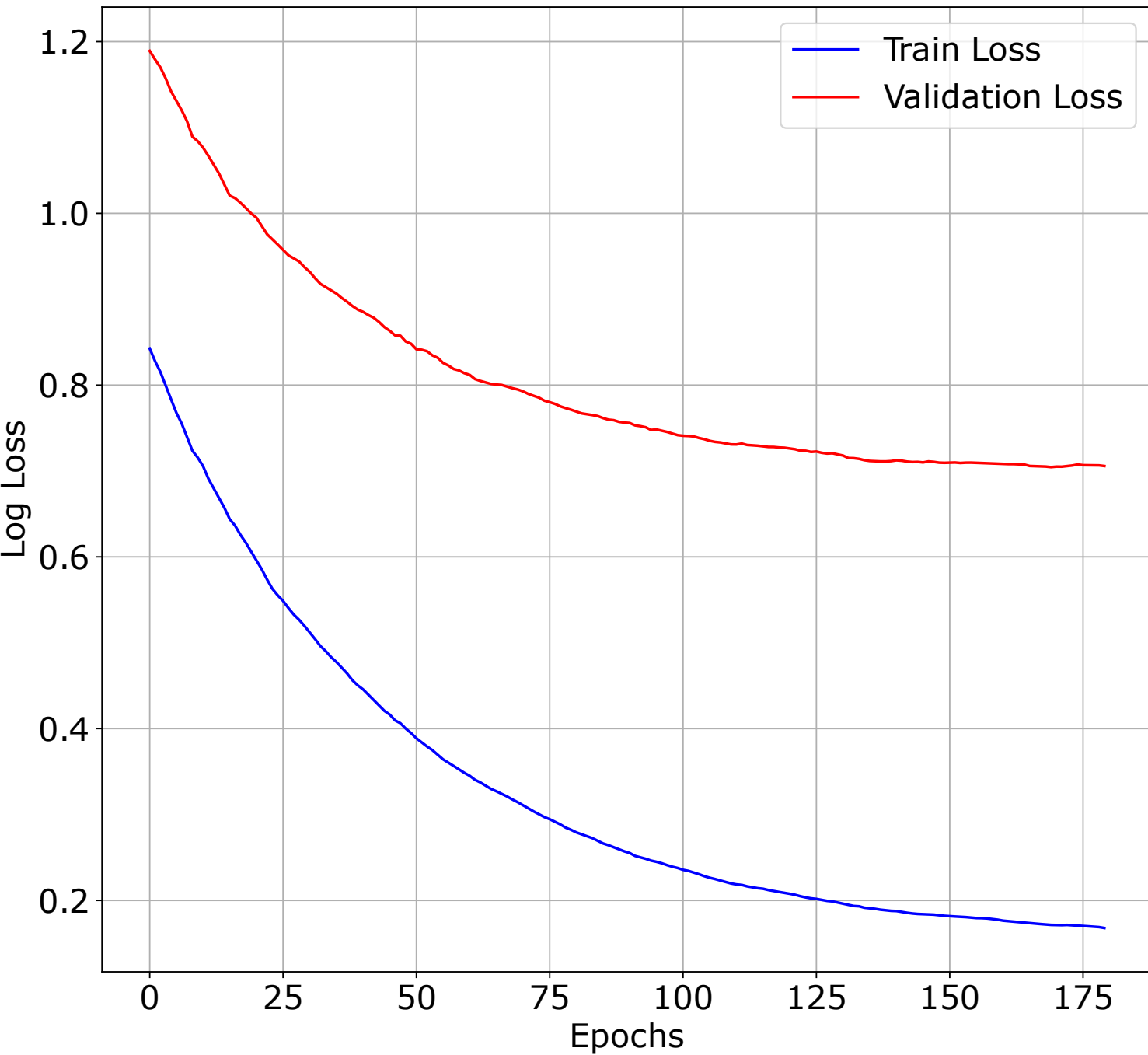

Supplement: Supplementary file 1 [file cancers-17-00214-s001.zip › Supplementary_Figure3.pdf]
